# John Stearne (1624–1669). Founder of the Royal College of Physicians of Ireland and first Professor of Medicine in Trinity College Dublin

**DOI:** 10.1007/s11845-025-03921-8

**Published:** 2025-03-06

**Authors:** Joseph Harbison

**Affiliations:** 1 Department of Medical Gerontology, School of Medicine, Trinity College Dublin, Dublin, Ireland; 2 Mercer’s Institute for Successful Ageing, St James’s Hospital, Dublin, Ireland; 3 Royal College of Physicians of Ireland, Dublin, Ireland

**Keywords:** Biography, Medical education, Medical history

## Abstract

John Stearne was the first Regius Professor of Medicine in Trinity College Dublin and founded the Fraternity of Physicians of Trinity Hall that later became the Royal College of Physicians in Ireland. He was born in Ardbraccan, County Meath in 1624 and was a great nephew of the Archbishop of Armagh and renowned scholar James Ussher who was his patron. He entered Trinity College in 1639 and was elected Scholar in 1641, before fleeing Dublin at the outbreak of the Confederate Wars later that year. He moved to Cambridge and studied medicine in Sidney Sussex College. After a short period practicing medicine in Bedfordshire, he returned to Trinity in 1651 and was appointed Professor of Medicine and College medicus. He later also became Professor of Hebrew and Professor of Law. He wrote six books and became a Senior Fellow of the College. In 1654, he established the Fraternity of Physicians with some other Dublin physicians in a disused building on Trinity Street. Stearne resigned his Fellowship and Professorship in 1659 but had them restored by 1662 following the Restoration of the King Charles II. The Royal College of Physicians of Ireland received its first royal charter in 1667. Stearne died in 1669 at the age of 44. This paper, written to celebrate the quatercentenary of his birth, discusses Stearne’s life and achievements in more detail particularly in the context of the political turmoil of the age and the important role of his extended family in the important events that occurred.

November 2024 is the start of the quatercentenary year of the birth of John Stearne, founder of the Royal College of Physicians of Ireland and first Professor of Physic in Trinity College Dublin. His standing as a key figure in the development of medicine in Ireland has meant that accounts of his life and career have been published to mark various anniversaries of both Colleges. Thomas Waugh Belcher (1865) [1], John Pentland Mahaffy (1912) [2] and Percy Kirkpatrick (1924) [3] have all published brief biographies of Stearne and others exist in sources such as the Dictionary of Irish Biography [4]. All of these have primarily used Stearne’s own writings to base their accounts in the absence of other sources. There is perhaps little to add beyond these works in relation to the key moments of his life, his publications and achievements although an up-to-date historiography is maybe overdue. We can conclude that Stearne was an intelligent, practical and stoical man who achieved a remarkable amount during a relatively short life in through tempestuous times. His legacy lies not in his writing, which serves more to help understand his life and motivations, than to impress by its intellectual content. He arrived at no insights or discoveries that have survived the centuries. His legacy is what he achieved to establish and develop the profession of medicine in Ireland. In this iteration of Stearne’s life it seems more valuable to put his story in the context of the world and the events of his experience, of which much more information has become available since Kirkpatrick’s paper written for his tercentenary.

John Stearne was born on the 26th of November 1624 in St Mary’s, Ardbraccan, a fortified Tudor mansion or castle. This was the episcopal seat of James Ussher who was Church of Ireland Bishop of Meath and Vice Chancellor of Trinity College Dublin. Stearne’s mother Mabel (nee Bermingham) was the daughter of Ussher’s sister Margaret, making Ussher her uncle. Stearne’s father, also named John, was a lawyer in the service of Theophilus Buckworth, Bishop of Dromore and he is described variously as being Buckworth’s ‘officer’ [4] or ‘chancellor’ [3]. The relationship between the Ussher and Buckworth families was likely close as Buckworth was married to another of Ussher’s sisters, Sarah, and their son was married to Mabel Ussher’s sister, and his own first cousin.

Within three months of Stearne’s birth, James Ussher was elevated to become Archbishop of Armagh and Primate of All Ireland adding to his influence within both Britain and Ireland. Ussher was a renowned historian, theologian and linguist [5]. He had strong connections to Trinity. He was one of the College’s first Scholars in 1596, became a Professor of Theology in 1612 and was Vice-Provost in addition to being Vice-chancellor in 1616. He married the daughter of a previous vice provost and left his extensive library to the University in his will. Trinity College ultimately reciprocated by naming a library after him in 2003. He was the first of a number of benefactors or patrons to substantially influence Stearne’s life. In the seventeenth century, this use and granting of patronage was an established, normal and even expected practice even in academia [6], and it is unlikely that Stearne would have achieved as much without his active canvassing of support from figures like Ussher and later Henry Cromwell. Indeed, in his book of Aphorisms, published later in his life [7], he wrote such epithets as ‘the good opinion of others about us is not to be neglected’, ‘each must have his own vocation and he must diligently pursue it’ and perhaps most remarkably ‘wealth is not to be neglected’ reflecting perhaps his approach to life and self-advancement.

Stearne spent his younger years living in the townland of Greenan in County Down where he reported that he received a liberal education under his schoolmaster Mr Burne. I could find no record of Mr Burne’s school but Greenan is about 30 km south of Dromore where the bishop’s seat was located which suggests that Stearne was a boarding student. In 1639, at the age of 14 years, he entered Trinity College Dublin. The College from Stearne’s time would be unrecognisable from that which stands today [8]. It was founded in 1592 on the site of an Augustinian Priory which had largely been demolished and rebuilt in red brick. Only the tower and spire of the priory church remained and these are visible on John Speed’s map from 1610 [9]. There is little left in modern Dublin that would be familiar to Stearne on his arrival in 1639 [10]. The population was around twenty thousand and although the centre of English rule, even following the 9 years’ war would still not have been recognised as ‘the capital’ far beyond the city or its pale. Many of the large public buildings, including both cathedrals, were dilapidated or even near derelict. One of the motivations for Thomas Smith, the Lord Mayor in the 1590s, to have the old Augustinian priory converted to a university was the increasing dereliction of the structure which had been pillaged for its valuable building materials [11].

The city had begun to recover from the effects of the Elizabethan war. While many of the houses were still built of wood, increasingly brick or stone was being used, at least in part, to reduce the risk of fire. Most of the houses were of only one or two stories but some were taller and grander. The last of the late sixteenth-century cage-work merchant’s houses were only demolished in the early nineteenth century [10]. The city had also begun to expand beyond its walls, in which there were now so many gates and breaches that they retained no real practicable defensive purpose. The city had expanded to the north side of the river, along Thomas’ Street to the west and down Patrick’s Street and on the road to St Stephen’s Church in the south. These roads had attracted informal occupants and habitations and civic authorities complained that squatters’ dwellings had begun to spring up along many of the other approach roads and in areas like the Bishops’ Liberties [10].

The College still lay outside the city in the flood-prone and less desirable land to the east. Its function of Trinity was still primarily to produce an educated Protestant clergy to serve in the education and conversion of the Irish. Although the curriculum would have extended into the humanities and classics, most of it would have been useful to its evangelical purpose, including philosophical, theological and rhetorical content. The college had awarded at least one Doctorate of Medicine (MD) before Stearne’s arrival [11] but may have been a unique event. Indeed, William Bedell, the Provost between 1627 and 1629, had lamented the lack of Professors of Law or Medicine and acknowledged it had been ‘an error to neglect the faculties of law and physics’ [12]. At this period, the University existed in a fairly consistently impecunious state that matched that of its host city.

Stearne performed well academically on his arrival in the college. In May 1641, he became a Scholar, although strictly speaking, the College was prohibited by parliament to elect anyone at that time. This first association with Trinity was soon to end with the outbreak in October of Irish Confederate Wars which was arguably the most destructive war in Irish history [13]. Relations between King Charles I and parliament in England had become increasingly strained and concessions made by the King to Catholics in return for support had caused disquiet both in the protestant population and church. Irish catholic nobles took advantage of the deteriorating political situation in Britain to reassert their rule and reclaim lands lost in the Elizabethan plantations. The war began with considerable sectarian bloodletting where perhaps 15,000 to 20,000 Protestants and several thousand Catholics died either through violence or resulting privation. This led to an exodus of many from the Protestant community to England, including the Provost of Trinity along with most of the Fellows and scholars including John Stearne.

It is thought that he initially went to stay with his great uncle Archbishop Ussher who was the incumbent Vice-Chancellor of Trinity and who had been in England at the outbreak of the rebellion. The King had supported Ussher with the income from the Diocese of Carlisle as the bishopric was vacant at the time.We don’t know how the rest of his family responded during this period, but his father’s employer Bishop Buckworth also fled to England. We know that his father survived the initial bloodletting, because was one of those who recorded the 1641 depositions from protestant survivors [14]. Through the good graces of Ussher, who was an acquaintance and correspondent of Samuel Ward, Master of Sidney Sussex College in Cambridge, Stearne was able to resume his studies there in July 1642. He soon graduated BA there, and in 1643 was elected a Fellow of Trinity College Dublin. In 1646, he was awarded an MA from Cambridge having completed the proscribed curriculum and he remained there until 1649.

We know that Stearne studied medicine in Cambridge, but it’s not clear that he obtained any specific medical qualification. In the mid seventeenth century, there would likely have been less than eighty studying medicine in Cambridge at any time amongst about six hundred total undergraduates and Sidney Sussex was one of the Colleges that particularly attracted them [15]. There does not seem to have been any systemic teaching of medicine in the University; Francis Glisson the Regius Professor of Physics never provided lectures [16], and there is little evidence of anatomical dissections being performed. The medical training received was largely theoretical and during this period, more than half the medical graduates of Cambridge spent time in Leiden to obtain some clinical instruction at the University there. Stearne does not appear to have been amongst them as he does not appear in the Leiden records which, unlike the Cambridge records of the time, are intact. In all probability, he would have studied the works of Hippocrates, Galen and Celsus and possibly those of more modern authors such as Vesalius, Harvey and Sanctorius but he never mentions them in his writings. There certainly would have been little training as to the practical application of the principles learned. Although the College of Physicians of London had been founded for more than a century, in the mid-seventeenth century, a formal qualification in medicine or actual clinical training was not compulsory to practice medicine in England.

Stearne reported that his time in Cambridge was the happiest in his life, and he would not have ‘cared to change it for all of the riches of the Persian King’. Another student who was an undergraduate in Emmanuel College while Stearne was studying there was Henry Cromwell, son of the local MP for Huntington, Oliver. Emmanuel and Sidney Sussex were considered the two most Calvinist colleges in Cambridge at the time and were geographically close to one another, so it is possible or even likely that the two students knew each other to some degree. Certainly, his relationship with Henry was of considerable benefit to Stearne in later years. In 1649, he left Cambridge for Oxford accompanying Seth Ward, a relative of Samuel’s. Stearne did not take up a sinecure at the University; however, his acquaintance with Ward, the newly appointed Savilian Professor of Astronomy, only allowed him to obtain temporary hostel accommodation. He soon moved to Bedfordshire to the north of London where it is assumed he began to practice medicine to support himself. It is unclear what precipitated Stearne’s departure from Cambridge. It may simply have been that he had completed his studies, had no academic position and had no reason to stay. He had previously had his Fellowship of Trinity College Dublin removed in 1648 by order of the ‘rump’ parliament. However, 1649 was also a momentous year in the history of both England and Ireland, and the events of the year almost certainly changed the course Stearne’s life. In January, Charles I was executed, effectively ending the Civil War in England and beginning the interregnum during which Oliver Cromwell became Lord Protector. In Ireland on August 2nd, a Parliamentarian force lead by Stearne’s cousin Michael Jones defeated the Duke of Ormond’s much larger joint Confederate and Royalist force at Rathmines, securing Dublin as a port for parliament. This victory facilitated the arrival of Oliver Cromwell with 15,000 troops to the city [13]. Tragically, it may also have facilitated the arrival of bubonic plague with the army which ultimately an estimated half of the population of the city over the next 18 months. The dead included the Provost of Trinity, Anthony Martin who had previously succeeded Ussher as Bishop of Meath. It may have been this event that motivated the Fellows to proceed with the appointment of a Public Professor of Physics whose role was also to act as *medicus* or physician to the senior fellows and provost of the College. It would not have harmed his cause that his cousin Henry Jones, brother to Michael and recently appointed Bishop of Clougher, had succeeded his uncle James Ussher as Vice chancellor of the University in 1646. Stearne was appointed to Trinity in October 1651 and had his fellowship restored with the support of Henry Cromwell who was now a senior commander of the Parliamentarian army in Ireland.

It is worth exploring how politics and religion influenced Stearne’s life and fortune to this point, as the two are inextricably linked at this period. It is to be emphasised that Stearne was unlikely to have been a passive player in the events affecting his life, but we have no correspondence or biography that gives any detail of his role in influencing them. In the early to mid-seventeenth century, the Church of Ireland was considerably more Calvinist in outlook and theology than the Church of England. Ussher was a liberal but orthodox Calvinist, and one assumes that Stearne’s upbringing reflected this, and he was a pious and observant protestant. Stearne’s only son, again called John, later became Dean of St Patrick’s Cathedral and, following his cousin Henry, Bishop of Clogher. Stearne’s writing, which is mainly philosophical, is consistent with his background but is also surprisingly non-sectarian. His predominant influences include the stoic philosophers Epictetus, Seneca and Marcus Aurelius, and he makes little reference to Jesus at all. Whilst the stoics, and it is reasonable to include Stearne amongst these, emphasised the importance of virtue and self-control above all, the Roman stoics, including Seneca and the recently rediscovered Marcus Aurelius, had a more worldly and pragmatic philosophical view which is reflected in Stearne’s own aphorisms and actions. Acquisition of wealth and position, which would have been seen as a vice by earlier stoics, was more acceptable to the Romans and making the best of one’s position was seen as a virtue. In speculating about Stearne’s political affiliation, it can be noted that Marcus Aurelius, considered by some to be 'the ideal ruler’, was in particular a figure of admiration by those of the Royalist faction in the Civil War [17]. Charles I had an equestrian sculpture of himself commissioned imitating one of the emperor’s on the Capitoline Hill in Rome.

In contrast, Stearne argued strongly in defence of a conventional Calvinist religious and philosophical view in his book criticising the Dutch philosopher Adrian Heeerboord [18], who was a teacher of, and influence on Baruch Spinoza. Stearne also quotes St Thomas Aquinas in his writings, which is perhaps a little unexpected from a Calvinist, and although he was clearly suspicious of Catholicism, particularly in the light of the events of 1641, there is no great condemnation of it in his works. He did however see himself as an outsider in Ireland. He describes the native Irish Catholics as ‘aborigines’ in his writing, but in the original sense of the word meaning indigenous. He clearly sees himself as from a different and separate stock than the native Irish catholic population.

James Ussher was clearly the largest influence on Stearne in his early years. Whilst Ussher was a Calvinist, and as such could be expected to be sympathetic to the parliament, he was in fact of the royalist faction because he felt that, having taken an oath to the King, he owed him loyalty. This was clearly a strain on him when the king made concessions to the Catholic faction in the 1630s and even more so when Royalist and rebel Catholic Confederate forces allied in Ireland in 1643. This loyalty to the king was shared by Samuel Ward [19], the master of Sidney Sussex who took Stearne in. He was another intellectual Calvinist who was loyal to the king, and his royalism ultimately led him to be arrested and incarcerated in 1642 by the parliamentarians. He died soon after his release in 1643 and was succeeded by Richard Minshull, a more conventional parliament supporting cleric with both Calvinist beliefs and the support of both Oliver Cromwell and his then commanding officer in the Parliamentary army Edward Montagu, Earl of Manchester.

The University of Cambridge was strongly Royalist at the beginning of the war, whereas the city and surrounding area supported parliament. One of Oliver Cromwell’s first acts in the war was to intercept a consignment of silver plate being sent by the University to the King to help fund his army. Thus, although he was accepted into the college by a royalist, for much of his stay in Cambridge, the head of his college was a parliamentarian. In contrast, Henry Rich the Chancellor of the University was a moderate Royalist who was ultimately executed in 1649 for treason. It is unclear which side if either that Stearne supported, but from his writing one could suspect that views tended towards the Royalist faction but were moderate and pragmatic. He accepted help and one must assume canvassed for support from both parliamentarian and royalist figures. This relative lack of partisanship was not uncommon, the assumption that everyone in England was either a die-hard ‘Cavalier’ or ‘Roundhead’ is false. Both Minshull and Montagu opposed the execution of Charles I and ultimately supported the restoration of Charles II. Both parliamentarians kept their positions as Master of Sidney Sussex and Chancellor of the University of Cambridge after the restoration of the Monarchy. Even Henry, Cromwell’s younger and probably more competent son, was allowed to keep most of his lands and property in Ireland and to retire peacefully following the coronation of the new king.

In October 1651, Stearne was reappointed a fellow of Trinity by the University Council and also became both medicus to the college and public Professor of Physic, the first in the College’s history. At this point, the Provost’s chair was vacant as a successor to Martin had not yet been selected. The Chancellor was still the defeated Duke of Ormond and, given that the city lay in Cromwell’s control and Ormond had fled to France, it is unlikely he had any influence in the appointment. This acknowledged, in 1652, Samuel Winter, another Cambridge graduate and supporter of Cromwell, became Provost and Stearne is recorded in the College register as Registrarus (Registrar) thus making him a Senior Fellow.

The city Stearne returned to had changed substantially since he left for Cambridge. While its population had transiently increased during the war consequent to the arrival of a few thousand displaced people and refugees, the 1649–1650 plague had killed nearly half the population, and a campaign of expelling Catholics from the city had begun, so the population may only have been a little over 10,000 in 1650. The population was augmented by the arrival of some English people and soldiers, but there must have been a substantial number of abandoned and derelict houses within the city [9].

Stearne prospered in Trinity following his appointment. In 1655, he received permission from the board to leave the hospital at night to attend to patients within the city. In 1656, following Winter’s insistence that all undergraduates be taught Hebrew and Greek, Stearne was appointed Professor of Hebrew in addition to his role as Professor of Physic. He did not take up the role until 1659 due to a dispute over salary that was eventually resolved following the intervention of Henry Cromwell. Around 1658, it is assumed he was awarded his MD by Trinity or at least he began signing himself as MD. Previous authors have suggested this may have been a degree awarded *ad eundem gradum* because of a prior Cambridge qualification [3] but there is no evidence for this. In November 1659, he resigned his Fellowship possibly because he married his wife Dorothy, and at that time, this marriage not permitted for Fellows. It is also probable that he foresaw the likely restoration of the Monarchy and holding a Fellowship and Professorship at the behest of the previous regime would not have been politically wise. At the restoration, Stearne was the second signatory of a letter or ‘Humble address and Petition’ by a group of students and faculty to the deposed Chancellor, the Duke of Ormonde, celebrating his return from exile, welcoming the reinstatement of the King and condemning the previous regime in the college [20]. Winter was forced out as Provost and Ormond was reinstated as Chancellor. In 1660 at the behest of the new King, Stearne was reappointed as a Senior Fellow and the same year received the degree of Doctor of Laws and became Professor of Law. In 1662, he was re-elected as Kings Professor of Physick.Since the 19th century such King's Professors are referred to as Regius.

Stearne’s shifting and pragmatic loyalties can also be seen in the dedications of his published books. The first ‘Animi Medela’ (1653) [21] is dedicated to Henry Cromwell who was newly appointed Chancellor of the College. The second ‘Thanatologia’ (1656) [22] was dedicated to the members of the now parliamentarian-leaning University Council, the third ‘Clarissimi Viri Adriani Heereboordi disputationum de concorsu examen’ (1660) [18] bears no dedication and his final two books ‘De Electione et Reprobatione’ (1662) [23] and ‘Aphorismi de Felicitate’ (1664) [7] were dedicated to the Duke of Ormond who had been reinstated as both college Chancellor and Lord Deputy of Ireland. His final work, edited by his former student and obituary writer Henry Dodwell, was published posthumously in 1672 [24]. Its topic is on the application of stoic philosophy in Christian theology, and its title has often erroniously been recorded as ‘De Destinatione’ or ‘about destination’ [3]. The correct tile is however De Obstinatione, meaning ‘about obstinacy’, which perhaps tells us more about Stearne’s personality and beliefs.

In 1604, the Corporation of Dublin had engaged a Mr Breddan to construct a Bridewell or prison for criminals and vagrants in the area called Hoggen Green between the City and College [25]. A dispute arose and the prison was never used for its intended purpose. Ultimately, the Irish Privy Council decided on a resolution and payment to be made to the builder; however, the Corporation were not pleased, and instead, allowed Trinity College to adopt it as a school or hall of residence for students which they named Trinity Hall. The Bridewell is visible on Speed’s 1610 map; however, this is not absolutely to scale. Superimposition of Speed’s [26] on later maps suggests that it was built roughly at the intersection of Trinity Street, named for the building, and Dame Lane.

The college renovated the building for students; however, it proved difficult to manage, and when the war began, the number of students entering Trinity dropped and the building fell into disuse except by some refugees displaced by the war [25]. With the restoration of order in the early 1650s, the Corporation moved to regain possession as it was not being used as agreed, and in 1654, Stearne, then a senior Fellow, offered to take on and restore the building so that it could be used for the teaching of medical students. Trinity board agreed and Stearne was joined by a number of other physicians in financially contributing to its restoration and conversion. A condition of the creation of this Fraternity of Physicians of Trinity Hall was that Stearne would live there and be President for life. He received dispensation from the College Board to do so, and his 1660 book ‘Clarissimi Viri’ is signed *ex aula Trinitatis* suggesting it was written there.

The motivations behind the foundation of the Faculty are probably multiple. The Royal College of Physicians of London had been founded in 1518 and in 1599 a College of Surgeons and Physicians in Glasgow in 1599. There was widespread acceptance of the need for a structure in which to train physicians and in particular to enable practical components of such training, such as anatomical dissection, to take place. There is no record of cadaveric dissections taking place in Trinity Hall during Stearne’s tenure as President but there are references made later in the century.

One must also take account of the conditions in the city at the time of the founding. There was no hospital still extant after the closure of St John’s and St Stephen’s in the 1540s [27]. Within Dublin, there was limited availability of medical care, much of which would have been delivered by apothecaries who had been accepted into the Barbers Guild of St Mary Magdalene founded in 1446 [28]. Thomas Smith, the Lord Mayor of Dublin at the founding of the College, was an apothecary.

Outside the larger towns, medical care may have been more informal such as by bone-setters or perhaps delivered by one of the hereditary Medical families common in Ireland and Scotland. Research into the training of these hereditary physicians has shown a level of practical and theoretical training comparable to that received by more conventionally trained physicians like Stearne. Study of surviving documents shows knowledge of European medical texts including translations of works from orthodox physicians. Finally, there was a burgeoning of less reputable providers of care in the mid to late seventeenth century with increased emergence of quack doctors, mountebanks and peddlers of patent cures [12]. So, the Fraternity, and later the College granted its first charter by Charles II in 1667, was a means of regulating training and care and, it must be acknowledged, also created what amounted to a cartel for the physicians who were members.

We know little about Stearne’s life as a physician except that he was clearly highly admired for his skill. He received special dispensations from the College that permitted him not only to reside outside the precinct but also not to attend morning religious services to allow him to attend his patients. None of his books strictly speaking relate to medicine although his first two books ‘Animi Medela’ and ‘Thanatologia’ do explore the nature of death and dying and the potential for prolonging life indefinitely. His exploration is more metaphysical than medical; however, he did write approvingly of the value of cold baths and the pain-relieving benefit of morphine. He also recommended the chewing of tobacco as a sedative [3].

Stearne died in November 1669, just before his 45th birthday. The cause of his death is unknown but he had time to correspond with Henry Dodwell, his former student and future Camden Professor of Hiistory at Oxford, before he died. He had four children, three daughters and a son, John, who later donated the Printing house to Trinity which still stands to this day.

Stearne was originally interred in the crypt of the old Chapel of Trinity. This was demolished in the 1790s and all the remains interred there, including one must assume Stearne’s, were gathered up reinterred in the tiny cemetery called “Challoner’s Corner” behind the new Chapel in front Square. His memorial stone, bearing the epitaph composed by Dodwell was moved to the front wall of the Chapel and remains the only such memorial in Front Square. It is very worn and hard to decipher but reads.

It is an accursed thing not to die.

Epictetus spake: to him hearkened.

JOHN STEARNE

M. & J. U. D. of the College of the Holy and Undivided Trinity at Dublin, Senior Fellow of the College of Physicians in the same place, First President, who was born at Ardbraccan November 26, 1624, died at Dublin November 18, 1669: Whose mortal trappings, hereafter to be put on again, are here laid by.

Philosopher, Physician, and consummate Theologian in one, Stearne, now nought, is resting in the ground.

No doubt unfriendly Death, to prove his authority, has divided into two parts what Nature brought forth as one.

But though thus now divided, Stearne will become one again and after the last day will pass in unity to immortality.

In conclusion, Stearne’s contribution to medicine, or to thought in general, does not come from his writing or publications. What we can observe from his short life is that he was able to adapt and prosper in challenging, and frequently dangerous times. He is described as erudite by a number of previous biographers and I see no reason to disagree, but I would add the word pragmatic. He made the best of what patronage he had and his achievements, the School of Medicine in Trinity and the Royal College of Physicians of Ireland have arguably been more positive and durable than those of any of his, at the time, more politically active or notable relatives.
